# Chemogenetic Regulation of CX3CR1-Expressing Microglia Using Gi-DREADD Exerts Sex-Dependent Anti-Allodynic Effects in Mouse Models of Neuropathic Pain

**DOI:** 10.3389/fphar.2020.00925

**Published:** 2020-06-19

**Authors:** Fumihiro Saika, Shinsuke Matsuzaki, Daichi Kobayashi, Yuya Ideguchi, Tomoe Y. Nakamura, Shiroh Kishioka, Norikazu Kiguchi

**Affiliations:** ^1^Department of Pharmacology, Wakayama Medical University, Wakayama, Japan; ^2^Department of Immunology, Niigata University Graduate School of Medical and Dental Sciences, Niigata, Japan; ^3^Faculty of Wakayama Health Care Sciences, Takarazuka University of Medical and Health Care, Wakayama, Japan

**Keywords:** allodynia, chronic pain, oxaliplatin, paclitaxel, spinal cord

## Abstract

Despite growing evidence suggesting that spinal microglia play an important role in the molecular mechanism underlying experimental neuropathic pain (NP) in male rodents, evidence regarding the sex-dependent role of these microglia in NP is insufficient. In this study, we evaluated the effects of microglial regulation on NP using Gi-designer receptors exclusively activated by designer drugs (Gi-DREADD) driven by the microglia-specific *Cx3cr1* promoter. For the Cre-dependent expression of human Gi-coupled M4 muscarinic receptors (hM4Di) in CX3C chemokine receptor 1-expressing (CX3CR1^+^) cells, R26-LSL-hM4Di-DREADD mice were crossed with CX3CR1-Cre mice. Mouse models of NP were generated by partial sciatic nerve ligation (PSL) and treatment with anti-cancer agent paclitaxel (PTX) or oxaliplatin (OXA), and mechanical allodynia was evaluated using the von Frey test. Immunohistochemistry revealed that hM4Di was specifically expressed on Iba1^+^ microglia, but not on astrocytes or neurons in the spinal dorsal horn of CX3CR1-hM4Di mice. PSL-induced mechanical allodynia was significantly attenuated by systemic (intraperitoneal, i.p.) administration of 10 mg/kg of clozapine N-oxide (CNO), a hM4Di-selective ligand, in male CX3CR1-hM4Di mice. The mechanical threshold in naive CX3CR1-hM4Di mice was not altered by i.p. administration of CNO. Consistently, local (intrathecal, i.t.) administration of CNO (20 nmol) significantly relieved PSL-induced mechanical allodynia in male CX3CR1-hM4Di mice. However, neither i.p. nor i.t. administration of CNO affected PSL-induced mechanical allodynia in female CX3CR1-hM4Di mice. Both i.p. and i.t. administration of CNO relieved PTX-induced mechanical allodynia in male CX3CR1-hM4Di mice, and a limited effect of i.p. CNO was observed in female CX3CR1-hM4Di mice. Unlike PTX-induced allodynia, OXA-induced mechanical allodynia was slightly improved, but not significantly relieved, by i.p. administration of CNO in both male and female CX3CR1-hM4Di mice. These results suggest that spinal microglia can be regulated by Gi-DREADD and support the notion that CX3CR1^+^ spinal microglia play sex-dependent roles in nerve injury-induced NP; however, their roles may vary among different models of NP.

## Introduction

Chronic pain is a serious problem that not only afflicts patients but also represents a substantial economic burden to the international community (Smith and Torrance, 2012). Neuropathic pain (NP), resulting from a lesion in the peripheral or central nervous system (PNS or CNS), is characterized by pain sensation in response to innoxious stimuli (allodynia) and increased sensitivity to pain (hyperalgesia) (Jensen and Finnerup, 2014). Moreover, treatment with anti-cancer agents [such as paclitaxel (PTX) and oxaliplatin (OXA)] has also been shown to induce NP (Colvin, 2019). Chemotherapy-induced neuropathic pain (CINP) markedly worsens the quality of life of cancer patients and often leads to chemotherapy discontinuation (Hershman et al., 2014). It is estimated that 7%–8% of the general population suffers from NP (van Hecke et al., 2014); however, the molecular and cellular mechanisms underlying this condition are poorly understood. Hence, there is a strong need for better insights into such mechanisms and the development of effective therapeutics based on these mechanisms.

Accumulating evidence suggests that an interaction between the immune system and the nervous system, including the brain, spinal cord, dorsal root ganglia, and peripheral nerves, largely contributes to the pathogenesis of NP (Ji et al., 2016). In the PNS, bone marrow-derived macrophages clearly accumulated in the damaged nerves and drive long-lasting inflammation underlying NP (Scholz and Woolf, 2007; Ji et al., 2016; Kiguchi et al., 2017). On the other hand, among the variety of spinal glial cells, microglia, the resident macrophages of the CNS responsible for its innate immunity, play a critical role in the development and maintenance of NP (Chen et al., 2018; Inoue and Tsuda, 2018). Several researchers have shown that spinal microglia become activated and secrete various inflammatory mediators, such as cytokines and chemokines, that sensitize pain-processing neurons in the spinal dorsal horn (SDH) (Milligan and Watkins, 2009; Ji et al., 2016). However, recent reports have demonstrated that spinal microglia are important in NP in male, but not in female, mice, suggesting sex-dependent roles for microglia in NP (Sorge et al., 2015; Rosen et al., 2017). Further investigations focusing on sex differences in NP pathogenesis are warranted to uncover the underlying mechanisms and develop effective therapeutics.

Designer receptors exclusively activated by designer drugs (DREADD) are genetically modiﬁed G-protein-coupled receptors (GPCRs). This technology represents a useful chemogenetic strategy, allowing researchers to gain control of Gi- or Gq-signaling pathways, remotely and noninvasively (Urban and Roth, 2015). The DREADD technology has been frequently used to regulate the activity of various types of neurons. Through application of selective ligands, clozapine-N-oxide (CNO) can stimulate or inhibit certain populations of glutamatergic, GABAergic or dopaminergic neurons (Dell’Anno et al., 2014; Koga et al., 2017; Zhang et al., 2020). In addition, the DREADD system has been used to regulate cellular activities of myeloid cells, breast cancer cells, and glial cells (Urban and Roth, 2015). Furthermore, Grace et al. have demonstrated that chemogenetic inhibition of spinal microglia *via* human Gi-coupled M4 muscarinic receptors (hM4Di) attenuates mechanical allodynia following peripheral nerve injury, while chemogenetic activation of spinal microglia *via* human Gq-coupled M3 muscarinic receptors (hM3Dq) induces mechanical allodynia in naïve rats using viral gene transfer (Grace et al., 2018). Nevertheless, state-dependent effects of Gi- or Gq-DREADD on intracellular signaling in microglia are still unclear, and evidence regarding the sex-dependent role of microglia in NP is insufficient because of the diversity of the experimental models used to study the condition.

In this study, we evaluated the sex-dependent effects of microglial regulation on NP caused by partial sciatic nerve ligation (PSL) and CINP, using Gi-DREADD driven by the microglia-specific *Cx3cr1* promoter (CX3CR1-hM4Di), in mice.

## Materials and Methods

### Mice

All animal experiments were approved by the Animal Research Committee of Wakayama Medical University and were carried out in accordance with the in-house guidelines for care and use of laboratory animals of Wakayama Medical University and the Ethical Guidelines of the International Association for the Study of Pain. R26-LSL-hM4Di-DREADD mice [B6N.129-Gt(ROSA)26Sor^tm1(CAG-CHRM4*,-mCitrine)Ute^/J; stock #026219] (Zhu et al., 2016) and CX3CR1-Cre transgenic (Tg) mice [Tg(Cx3cr1-cre)MW126Gsat/Mmucd; stock #036395] were purchased from the Jackson Laboratory and Mutant Mouse Resource & Research Centers (MMRRC), respectively. R26-LSL-hM4Di-DREADD mice were maintained as heterozygous or homozygous genotype. For the Cre-dependent expression of the Gi-DREADD system in the *Rosa26* locus in CX3CR1-expressing (CX3CR1^+^) cells, R26-LSL-hM4Di-DREADD mice were crossed with CX3CR1-Cre mice. Subsequently, 6–12-week-old mice heterozygous for ROSA26 and CX3CR1-Cre were used for the experiments. All mice were housed in groups of 5–6 in plastic cages at controlled temperature (23°C–24°C) and humidity (60%–70%), and the environment was maintained on a 12-h dark/light cycle, with free access to standard food and water.

### Drug Administration

Paclitaxel (TAXOL^®^ Injection; Bristol-Myers Squibb Company, New York, NY, USA) and oxaliplatin (ELPLAT^®^ i.v. infusion solution; Yakult Honsha Co., Ltd, Tokyo, Japan) were diluted in 5% glucose solution. Clozapine N-oxide (CNO: Enzo Life Sciences, Farmingdale, NY, USA) was dissolved in sterile water and diluted as needed. CNO was administered intraperitoneally (i.p.) at a volume of 0.1 ml/10 g body weight to awake mice or intrathecally (i.t.) at a volume of 5 μl to isoflurane-anesthetized mice, as previously described (Kiguchi et al., 2020). Under isoflurane anesthesia, mice were secured by a firm grip on the pelvic girdle, and drugs were injected by lumbar puncture between the L5 and L6 vertebrae using a 30-gauge needle fitted with Hamilton microsyringe.

### Neuropathic Pain Models

#### Partial Sciatic Nerve Ligation (PSL) Model

The mice were subjected to PSL as previously described (Seltzer et al., 1990; Kiguchi et al., 2018). Briefly, under isoflurane anesthesia, the left common sciatic nerve (SCN) of each mouse was exposed at the mid-thigh level through a small skin incision on one side, hereafter indicated as ipsilateral. Approximately one-third of the SCN thickness was tightly ligated with a silk suture (No. 1; Natsume Seisakusho, Tokyo, Japan); then, the muscle and skin layers were closed with sutures and the surgical area was sterilized with povidone–iodine. The untreated right limb is indicated as contralateral.

#### Chemotherapy-Induced Neuropathic Pain (CINP) Models

Paclitaxel (4 mg/kg/day), oxaliplatin (5 mg/kg/day), or vehicle (Veh; 5% glucose solution) was administered i.p. to the mice at a volume of 0.1 ml/10 g body weight four times every second day (on days 0, 2, 4, and 6).

### Immunohistochemistry

The mice were deeply anesthetized with pentobarbital (100 mg/kg, i.p.) and transcardially perfused with ice-cold phosphate-buffered saline (PBS), followed by 4% (w/v) paraformaldehyde/phosphate buffer solution. Then, the lumbar spinal cord (L4–5) or the SCN was dissected, post-fixed in 4% paraformaldehyde/phosphate buffer solution, and put overnight in a 30% (w/v) sucrose/PBS solution at 4°C for cryoprotection. The tissue was then embedded in freezing optimal cutting temperature compound (Sakura, Tokyo, Japan). Subsequently, the specimens were longitudinally cut into 30 μm (spinal cord)- or 15 μm (SCN)-thick sections with a cryostat (Leica Microsystems, Wetzlar, Germany). The sections were treated with PBS containing 0.1% Triton X-100 (PBST) for 1 h and then blocked with Blocking One Histo (Nacalai Tesque, Inc., Kyoto, Japan) at 15°C–25°C for 5–10 min. They were then incubated with primary antibodies against hemagglutinin (HA) epitope-tag (mouse monoclonal, 1:250; BioLegend, San Diego, CA, USA), Iba1 (for microglia; rabbit polyclonal, 1:500; Wako, Japan), GFAP (for astrocytes; rabbit polyclonal, 1:500; Proteintech, Rosemont, IL, USA), NeuN (for neurons; rabbit monoclonal, 1:500; Millipore, Billerica, MA, USA), and F4/80 (for macrophages; rat monoclonal, 1:200; Cederlane, Burlington, Canada) in Blocking One Histo/PBST at 4°C overnight. Subsequently, the sections were rinsed in PBST and incubated with fluorescence-conjugated secondary antibodies (1:200; Abcam, Cambridge, UK) at 15°C–25°C for 2 h. They were then rinsed in PBS and incubated with Hoechst 33342 (1:1000; Invitrogen, Carlsbad, CA, USA) for 10 min at room temperature in the dark. Finally, the sections were washed with PBS (10 min), mounted on glass slides, and covered with a cover slip with PermaFluor (Thermo Fisher Scientific, Waltham, MA, USA). Fluorescence images were detected using a confocal laser scanning microscope (Carl Zeiss, Oberkochen, Germany).

### Behavioral Testing

von Frey test: To evaluate mechanical allodynia, the 50% paw withdrawal threshold was determined through the von Frey test, in accordance with a previously described method (Chaplan et al., 1994; Saika et al., 2019). Briefly, the mice were individually placed on a metal mesh (5 × 5 mm) grid floor and covered with an opaque acrylic box. Before the test, the mice were habituated to the experimental environment for at least 3–4 h. On the test day, after adaptation for 2-3 h, calibrated von Frey filaments (North Coast Medical, Inc., Gilroy, CA, USA) were applied to the middle of the plantar surface of the hind paw through the bottom of the mesh floor. The filament set used in this study consisted of nine calibrated von Frey filaments—0.02, 0.04, 0.07, 0.16, 0.4, 0.6, 1.0, 1.4, and 2.0 g. In the paradigm of the up–down method, the test always started with the application of 0.4 g filaments. Quick withdrawal, shaking, biting or licking of the stimulated paw were regarded as positive paw withdrawal response. In the absence of a paw withdrawal response to the selected force, the next stronger stimulus was applied. In the presence of paw withdrawal, the next weaker stimulus was chosen. In accordance with Chaplan et al.’s procedure, after the response threshold was first crossed (the two responses straddling the threshold), four additional stimuli were applied. Based on the responses to the von Frey filaments series, the 50% paw withdrawal threshold was calculated according to the method described by Dixon (Dixon, 1980).

Rotarod test: To assess motor function, the Rotarod apparatus (Panlab, Barcelona, Spain) was used. A few days before the test, the mice were pre-trained in order to habituate them to the rod, rotating at various speeds (5, 10, and 15 rpm). The mice were placed on the rod facing the opposite direction to the rotation and ambulated until they fell from the rod or the maximum observation time had elapsed. The maximum observation time was 180 s. For all experiments, the latency and rotational velocity at which the animal fell from the Rotarod were recorded. Following training for a few days, the mice rested for 1 day and were then tested (three trials per day) on the rod rotating at various speeds (10, 15, 20 rpm), with 10-15 min interval between each trial. The mean time spent on the Rotarod apparatus was measured on each velocity (Pre-test). After the Pre-test phase, the mice received a single i.p. administration of CNO (10 mg/kg). The test was repeated 24 h after administration (Post-test).

### Statistical Analysis

Data are presented as mean ± standard error of the mean (S.E.M.). Statistical analyses were performed using Student’s t-test, one-way analysis of variance (ANOVA) followed by Tukey’s multiple comparison test, or two-way ANOVA followed by Bonferroni’s multiple comparison test, as appropriate. P-values less than 0.05 were considered statistically signiﬁcant.

## Results

### hM4Di Expression in Spinal Microglia of CX3CR1-hM4Di Mice

**Figure 1:**
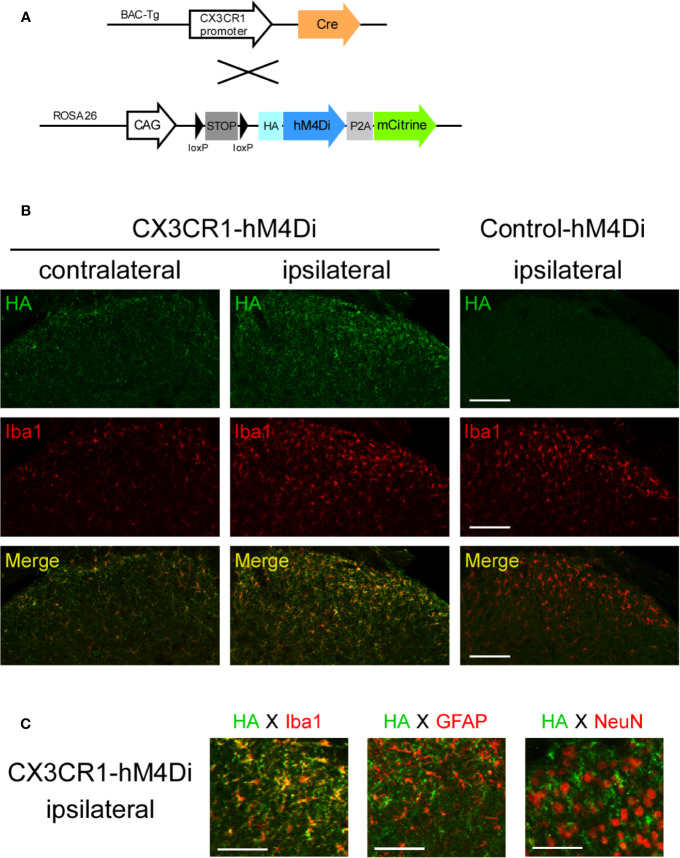
hM4Di expression in spinal microglia of CX3CR1-hM4Di mice. **(A)** Scheme of Cre-dependent expression of hM4Di in CX3CR1^+^ cells by crossing CX3CR1-Cre (Tg) mice with R26-LSL-hM4Di-DREADD mice (Control-hM4Di). Following Cre-mediated removal of an upstream floxed-STOP cassette, the HA-tagged hM4Di and mCitrine were expressed in CX3CR1^+^ cells. **(B, C)** The mice were subjected to partial sciatic nerve ligation (PSL), and the lumbar spinal cord (L4-5) was dissected on day 7 after PSL. Expression of HA-tagged hM4Di in the spinal dorsal horn (SDH) was visualized by immunohistochemistry. **(B)** Expression of HA-tagged hM4Di in the SDH of CX3CR1-hM4Di mice. Scale bars = 100 μm. **(C)** Localization of HA-hM4Di in Iba1^+^ microglia, but not GFAP^+^ astrocytes or NeuN^+^ neurons. Scale bars = 50 μm.

First, we evaluated the effects of chemogenetic modulation of CX3C chemokine receptor 1-expressing (CX3CR1^+^) cells on PSL-induced NP using Cre-dependent Gi-DREADD. For the Cre-dependent expression of hM4Di in CX3CR1^+^ cells, R26-LSL-hM4Di-DREADD (Control-hM4Di) mice were crossed with CX3CR1-Cre mice (**Figure 1A**). Following Cre-mediated removal of an upstream floxed-STOP cassette, the expression of HA-tagged hM4Di was observed using an antibody against HA by immunohistochemistry. Iba1^+^ microglia were markedly increased in the ipsilateral compared to the contralateral side of the SDH in both male Control-hM4Di and CX3CR1-Cre/R26-LSL-hM4Di-DREADD (CX3CR1-hM4Di) mice on day 7 after PSL. HA-hM4Di was highly expressed in the SDH of CX3CR1-hM4Di mice, but not in the SDH of Control-hM4Di mice (**Figure 1B**). In CX3CR1-hM4Di mice, HA-hM4Di overlapped with Iba1; however, it was not localized in GFAP^+^ astrocytes or NeuN^+^ neurons (**Figure 1C**). Additionally, F4/80^+^ macrophages were clearly accumulated in the injured SCN in both male Control-hM4Di and CX3CR1-hM4Di mice on day 7 after PSL. HA-hM4Di was also expressed in macrophages of CX3CR1-hM4Di mice, but not in macrophages of Control-hM4Di mice (**Figure S1**).

### Relief of Mechanical Allodynia After PSL by Systemic CNO in Male, but Not in Female CX3CR1-hM4Di Mice

**Figure 2:**
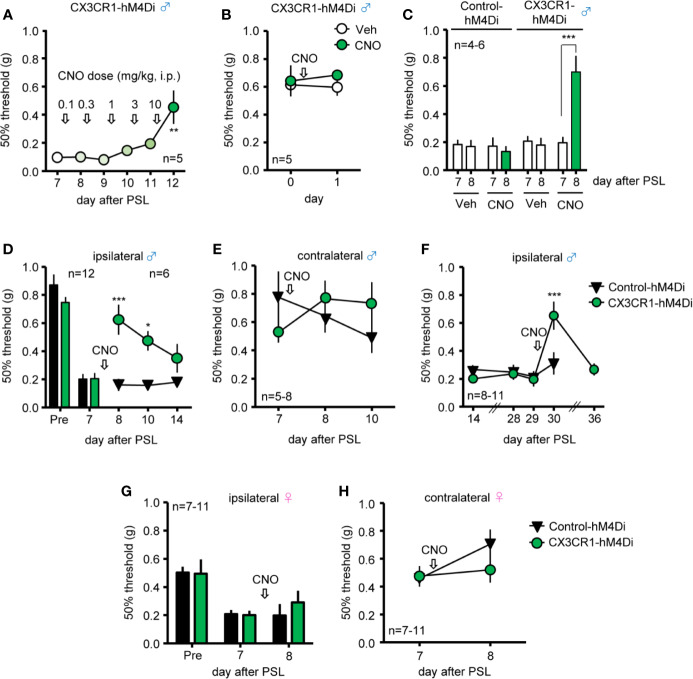
Relief of mechanical allodynia after PSL by systemic CNO in male CX3CR1-hM4Di mice. The mice were subjected to PSL, and CNO was intraperitoneally (i.p.) administered as follows: **(A)** Increasing doses of CNO (0.1-10 mg/kg, i.p.) were administered daily from days 7 to 11 after PSL, and pain assessment was performed the next day after each administration. **(B–H)** CNO was i.p. administered after pain assessment as indicated by arrows. The 50% paw withdrawal threshold was assessed by the up–down method using the von Frey test. **(A)** Effects of different doses of CNO (0.1-10 mg/kg) on mechanical allodynia after PSL were evaluated 24 h after each CNO administration (before next doses) in male CX3CR1-hM4Di mice. **(B)** No effect of CNO (10 mg/kg) on 50% paw withdrawal threshold in naïve male CX3CR1-hM4Di mice. **(C)** Relieving effect of CNO (10 mg/kg) on PSL-induced mechanical allodynia in male CX3CR1-hM4Di mice. **(D, E)** Effect of CNO (10 mg/kg) on the 50% paw withdrawal threshold in the ipsilateral and contralateral side of male CX3CR1-hM4Di and Control-hM4Di mice after PSL. **(F)** Prolonged relieving effect of CNO (10 mg/kg) on mechanical allodynia in male CX3CR1-hM4Di and Control-hM4Di mice after PSL. **(G, H)** Effect of CNO (10 mg/kg) on the 50% paw withdrawal threshold in the ipsilateral and contralateral side of female CX3CR1-hM4Di and Control-hM4Di mice after PSL. Data are presented as mean ± S.E.M. n = 5-11. p***< 0.001, p** < 0.01, p* < 0.05 vs day 7 or day 29 before CNO, or Control-hM4Di.

To determine whether induction of Gi-DREADD in CX3CR1^+^ cells affects PSL-induced mechanical allodynia in male mice, different doses of CNO (0.1-10 mg/kg, i.p.) were administered daily, from days 7 to 11 after PSL, and pain assessment was performed the day after each administration. PSL-induced mechanical allodynia was significantly attenuated by 10 mg/kg of CNO (**Figure 2A**), but neither CNO (10 mg/kg, i.p.) nor Veh altered the 50% withdrawal threshold in naive male CX3CR1-hM4Di mice (**Figure 2B**). PSL-induced mechanical allodynia was significantly prevented by single administration of CNO (10 mg/kg, i.p.) compared to administration of Veh in male CX3CR1-hM4Di mice, but not in male Control-hM4Di mice (**Figure 2C**). In both male CX3CR1-hM4Di and Control-hM4Di mice, CNO (10 mg/kg, i.p.) did not affect the time on the Rotarod at 10, 15, and 20 rpm on day 1 after administration, indicating no motor dysfunction due to CNO (10 mg/kg) (**Figure S2**). Single administration of CNO (10 mg/kg, i.p.) on day 7 after PSL transiently relieved mechanical allodynia (days 8-10), but the anti-allodynic effect disappeared within 7 days after CNO administration (day 14) in the ipsilateral side of male CX3CR1-hM4Di mice (**Figure 2D**). The 50% withdrawal threshold showed no significant difference in the contralateral side (**Figure 2E**). Moreover, even on day 28 after PSL, PSL-induced mechanical allodynia in the ipsilateral paw was significantly suppressed by CNO (10 mg/kg, i.p.) in male CX3CR1-hM4Di, but not in male Control-hM4Di mice (**Figure 2F**). However, CNO (10 mg/kg, i.p.) had no significant effect on the 50% withdrawal threshold in the ipsilateral or contralateral side of female CX3CR1-hM4Di mice (**Figures 2G, H**).

### Relief of Mechanical Allodynia After PSL by Intrathecal CNO in Male, but Not in Female CX3CR1-hM4Di Mice

**Figure 3:**
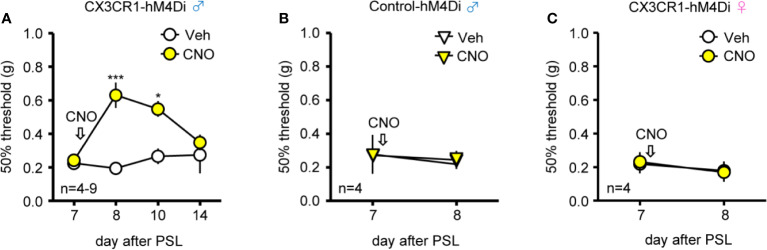
Relief of mechanical allodynia after PSL by intrathecal CNO in male CX3CR1-hM4Di mice. The mice were subjected to PSL, and CNO (20 nmol) or Veh was intrathecally (i.t.) administered after pain assessment as indicated by arrows. The 50% paw withdrawal threshold was assessed by the up–down method using the von Frey test. **(A**, **B)** Effect of CNO on the 50% paw withdrawal threshold in the ipsilateral side of male CX3CR1-hM4Di and Control-hM4Di mice after PSL. **(C)** No effect of CNO on mechanical allodynia in the ipsilateral side of female CX3CR1-hM4Di mice after PSL. Data are presented as mean ± S.E.M. n = 4-9. p***< 0.001, p* < 0.05 vs Veh.

Next, to clarify the effects of Gi-DREADD in spinal CX3CR1^+^ microglia under PSL-induced mechanical allodynia, CNO (20 nmol) or Veh was i.t. administered in both CX3CR1-hM4Di and Control-hM4Di mice. Single administration of CNO (20 nmol, i.t.) on day 7 after PSL significantly relieved PSL-induced mechanical allodynia compared to administration of Veh (days 8-10) in male CX3CR1-hM4Di but not Control-hM4Di mice; however, the anti-allodynic effect of i.t. CNO disappeared 7 days after CNO administration (day 14) in male CX3CR1-hM4Di mice (**Figures 3A, B**). In contrast, i.t. administration of CNO had no effect on PSL-induced mechanical allodynia in female CX3CR1-hM4Di mice (**Figure 3C**).

### Effects of CNO on PTX- or OXA-Induced CINP in CX3CR1-hM4Di Mice

**Figure 4:**
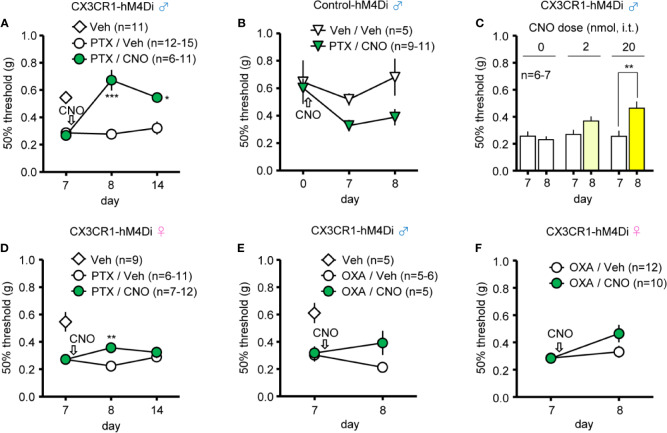
CNO effect on chemotherapy-induced neuropathic pain (CINP) caused by paclitaxel (PTX) and oxaliplatin (OXA) in CX3CR1-hM4Di mice. PTX (4 mg/kg) or OXA (5 mg/kg) was i.p. administered to mice four times every second day (on days 0, 2, 4, and 6), and CNO or Veh was administered after pain assessment as indicated by arrows. The 50% paw withdrawal threshold was assessed by the up–down method using the von Frey test. **(A**, **B)** Effect of CNO (10 mg/kg, i.p.) on the 50% paw withdrawal threshold in male CX3CR1-hM4Di and Control-hM4Di mice after PTX administration. **(C)** Dose-dependent effect of CNO (2 or 20 nmol, i.t.) on PTX-induced mechanical allodynia in male CX3CR1-hM4Di mice. **(D)** Effect of CNO (10 mg/kg, i.p.) on PTX-induced mechanical allodynia in female CX3CR1-hM4Di mice. **(E, F)** Effect of CNO (10 mg/kg, i.p.) on OXA-induced mechanical allodynia in male and female CX3CR1-hM4Di mice. Data are presented as mean ± S.E.M. n = 5-12. p***< 0.001, p** < 0.01, p* < 0.05 vs day 7 before CNO, or Veh.

Finally, we investigated whether induction of Gi-DREADD in CX3CR1^+^ cells can relieve CINP. Repetitive i.p. administration of PTX in male CX3CR1-hM4Di and Control-hM4Di mice caused mechanical allodynia on day 7 compared to administration of Veh (5% glucose solution) (**Figure 4A**). Single administration of CNO (10 mg/kg, i.p.) on day 7 relieved mechanical allodynia by PTX on the following day (day 8), and the anti-allodynic effect persisted for 7 days after CNO administration (until day 14) in male CX3CR1-hM4Di, but not in Control-hM4Di mice (**Figures 4A, B**). Moreover, single administration of CNO (20 nmol, i.t.) on day 7 significantly relieved PTX-induced mechanical allodynia as well (**Figure 4C**). In female CX3CR1-hM4Di mice also, single administration of CNO (10 mg/kg, i.p.) on day 7 attenuated PTX-induced mechanical allodynia on the following day (day 8), but this effect disappeared 7 days after CNO administration (**Figure 4D**). Unlike the PTX-induced effect, OXA-induced mechanical allodynia was slightly improved, but not significantly relieved, by single administration of CNO (10 mg/kg, i.p.) in both male and female CX3CR1-hM4Di mice (**Figures 4E, F**).

## Discussion

In this study, we provided several novel findings. CX3CR1^+^ microglia could be regulated by Gi-DREADD in mice, and PSL-induced mechanical allodynia was relieved by CX3CR1-Gi-DREADD, regardless of time after injury, in male but not in female mice. Moreover, the relieving effect of CX3CR1-Gi-DREADD on PTX-, but not OXA-induced allodynia, was dominantly observed in male compared to female mice. These lines of evidence illustrate model- and sex-dependent roles of spinal microglia in the pathophysiology of NP.

Among different microglial promoters, the *Cx3cr1* promoter system is frequently used to implement microglia-specific gene manipulation in mice. To date, different CX3CR1-Cre lines have been developed and widely used in a number of studies, focusing on the roles of microglia in the CNS (Parkhurst et al., 2013; Peng et al., 2016). However, it is also reported that some CX3CR1-Cre lines have a possibility for leakage of Cre expression into other cells, such as neurons and astrocytes (Hwang et al., 2017; Zhang et al., 2018). Zhao et al. demonstrated that CX3CR1 expression was neither observed in neurons nor in astrocytes in CX3CR1-Cre mice obtained from MMRRC (#036395) and concluded that the line was microglia-specific (Zhao et al., 2019). In agreement with this, our data also showed that hM4Di was expressed by Iba1^+^ microglia, but not GFAP^+^ astrocytes and NeuN^+^ neurons in the SDH of CX3CR1-hM4Di mice. On the other hand, hM4Di expression was also observed in F4/80^+^ macrophages in the PNS of CX3CR1-hM4Di mice. Given that macrophages and microglia share several common systems and molecules including CX3CR1 (Greter et al., 2015), it is expected that hM4Di is also expressed in not only spinal microglia but also peripheral macrophages.

CNO has been used as a selective ligand to induce Gi- and Gq-DREADDs in neurons. When CNO binds to hM4Di, a canonical Gi-protein-dependent pathway causes membrane hyperpolarization (Armbruster et al., 2007). Even though the molecular mechanisms of Gi-DREADD have been well documented in neurons (Urban and Roth, 2015), the effects of Gi-DREADD in non-neuronal cells are poorly understood. Regarding microglia, only two groups of researchers demonstrated that induction of Gi-DREADD may attenuate the activation of microglia with inflammatory phenotypes (Grace et al., 2018; Coleman et al., 2020). Nevertheless, detailed mechanisms and state-dependent effects of Gi- or Gq-DREADD on intracellular signaling in microglia are still unclear. It is important that we also confirmed that hM4Di-signaling in CX3CR1^+^ microglia can exert an inhibitory regulation through the Gi-DREADD system, resulting in NP relief in mice. Molecular-based mechanisms of microglial regulation by Gi- and Gq-DREADD need to be further investigated in the future.

Gomez et al. reported that systemically-administered CNO is converted to clozapine, which produces non-specific suppressive effects on locomotor activity in mice (Gomez et al., 2017); this suggests that it is important to carefully investigate the effects of CNO *in vivo*. Therefore, we compared the effect of CNO on CX3CR1-hM4Di mice with that on Control-hM4Di mice in all experiments. Unlike i.t. CNO administration (20 nmol), i.p. CNO injection (1 mg/kg) slightly decreased the locomotor activity of the mice, and higher doses of i.p. CNO (3-10 mg/kg) caused sedation over several hours after administration in both male and female CX3CR1-hM4Di mice. Given that CNO (10 mg/kg, i.p.) did not cause such sedative effects in Control-hM4Di mice, the induction of Gi-signaling in CX3CR1^+^ cells may elicit unfavorable behavioral effects during acute periods in both sexes. Nevertheless, we did not observe motor dysfunction 24 h after CNO administration (**Figure S2**) in CX3CR1-hM4Di mice, and the mechanical threshold of naïve CX3CR1-hM4Di mice was not altered by i.p. CNO injection. Therefore, to evaluate the selective roles of Gi-DREADD on CX3CR1^+^ cells on mechanical allodynia, behavioral testing was conducted 24 h after CNO administration, regardless of the delivery route.

It is well known that microglial activation in the SDH is linked to several types of NP in rodents (Chen et al., 2018; Inoue and Tsuda, 2018). In case of peripheral nerve injury, the damaged neurons send excitatory signals to the spinal microglia *via* chemical substances, such as ATP and chemokines; these, in turn, cause microglial proliferation and upregulation of inflammatory cytokines, such as TNF-α, IL-1β, and IL-6, resulting in NP (Milligan and Watkins, 2009; Zhao et al., 2017). Given that activated microglia largely affect neuronal and astrocytic activity under NP conditions, it is pivotal to determine disease- and sex-specific roles of microglia in NP. A large body of evidence indicates that inhibition of microglial activation by small molecule compounds, such as minocycline (Raghavendra et al., 2003; Ledeboer et al., 2005; Mei et al., 2011) and propentofylline (Tawfik et al., 2007), can prevent inflammatory responses and NP. Furthermore, also ablation of activated microglia by pharmacological or genetic approaches demonstrated that these cells significantly contribute to the NP condition (Sorge et al., 2015; Peng et al., 2016). However, in terms of specificity or time dependency, the current evidence is insufficient to uncover the complicated roles of microglia in NP. Importantly, our results clearly showed that both systemic (i.p.) and local (i.t.) administration of CNO significantly relieved PSL-induced mechanical allodynia in male CX3CR1-hM4Di mice, which is consistent with the results of previous studies (Sorge et al., 2015).

Sex difference in pain is clinically often observed, and the proportion of male patients suffering from chronic pain is lower than that of female patients (Mogil, 2012). However, to date, male animals have traditionally been used in the majority of laboratory pain studies. Mechanical allodynia develops similarly in both male and female mice after nerve injury, whereas there are significant differences in NP mechanisms between male and female (Boccella et al., 2019; Del Rivero et al., 2019; Inyang et al., 2019). In particular, the neuro-immune interaction seems to be one of the causes for sex difference in pathological pain (Rosen et al., 2017). Sorge et al. demonstrated that i.t. administration of glial inhibitors or microglia-targeting toxins exerted significant inhibitory effects on mechanical allodynia following nerve injury in male, but not in female mice, suggesting male-selective roles of microglia in NP (Sorge et al., 2015). Moreover, inhibitors of several microglia-related proteins, such as Toll-like receptor 4, brain-derived neurotrophic factor (BDNF), and purinergic P2X4 receptor (P2X4R) also produced sex-dependent effects on NP (Sorge et al., 2015). Notably, P2X4R is upregulated on microglia after nerve injury in male, but not in female mice, and the activation of P2X4R causes synthesis and release of BDNF through p38-mitogen-activated protein kinase, suggesting that P2X4R might be responsible for the sex differences in NP (Trang et al., 2009; Mapplebeck et al., 2016). In contrast to P2X4R, expression levels of CX3CR1 in the SDH after nerve injury were similar between in male and female mice (Taves et al., 2016), suggesting that Gi-DREADD might be commonly induced in both sexes. In consistent with these findings, we found that induction of Gi-DREADD in CX3CR1^+^ cells, by either i.p or i.t. CNO administration, exerted relieving effects on PSL-induced mechanical allodynia in male CX3CR1-hM4Di but not female mice, supporting the notion that microglial activation plays a pivotal role in the pathogenesis of NP only in male mice.

Since lumbar puncture i.t.-administered agents are known to distribute in the dorsal root ganglion (DRG) region (Kawasaki et al., 2008; Alessandri-Haber et al., 2009), it is difficult to rule out the possibility that CNO also affected other cells at this level. Based on the critical role of the macrophages infiltrating the peripheral nerves in NP (Thacker et al., 2007; Kiguchi et al., 2017; Saika et al., 2019), Gi-DREADD may also be induced in CX3CR1^+^ macrophages after i.p. or i.t. CNO administration. Recently, Yu et al. demonstrated that the depletion of DRG macrophages prevented nerve injury-induced mechanical allodynia not only in male but also in female mice (Yu et al., 2020). Given the sex-independent role of DRG macrophages, we hypothesized that the relieving effects of CX3CR1-Gi-DREADD on NP in male mice were mainly exerted *via* inhibitory regulation of CX3CR1^+^ microglia. Nevertheless, as several reports suggest sex-specific roles of peripheral immune cells (Luo et al., 2019), further studies are required to clarify common molecular mechanisms involved in NP.

CINP is the dose-limiting side effect of anti-cancer agents, such as PTX and OXA, and remains a challenging clinical problem. Previous reports clarified that PTX treatment induced microglial activation in the SDH, leading to NP that is attenuated by minocycline (Burgos et al., 2012; Pevida et al., 2013). Consistently, we found that i.p. or i.t. CNO administration reversed PTX-induced mechanical allodynia in male CX3CR1-hM4Di mice. Although CX3CR1-Gi-DREADD slightly improved mechanical allodynia also in female mice, the degree of improvement in this case was clearly lower than that in male mice. These results indicate that CX3CR1^+^ microglia play an important role in PTX-induced mechanical allodynia as well, at least in male mice. Unlike in the case of PTX, CX3CR1-Gi-DREADD did not show any significant preventive effect on OXA-induced mechanical allodynia in both sexes. Based on several cases in the literature regarding the mechanisms of OXA-induced NP (Di Cesare Mannelli et al., 2013; Robinson et al., 2014; Kerckhove et al., 2017), spinal microglia may not significantly contribute to OXA-induced mechanical allodynia. Given that astrocytes widely contributed to different types of CINP, common regulatory mechanisms in the CNS may also exist between PTX- and OXA-induced NP (Sisignano et al., 2014). Although there are controversial reports regarding the involvement of spinal microglia in PTX- or OXA-induced NP, our results emphasize the model- and sex-specific contribution of CX3CR1^+^ microglia to this condition; this is also supported by evidence obtained using microglial inhibitors, such as minocycline and propentofylline.

Collectively, we clearly demonstrated that chemogenetic regulation of CX3CR1^+^ microglia using Gi-DREADD exerted sex-dependent (i.e., male-selective) relieving effects on nerve injury-induced NP in mice. In contrast, CX3CR1-Gi-DREADD also prevented CINP caused by PTX, but not OXA, in male mice, suggesting that the contribution of spinal microglia to CINP may be agent dependent. Given that several gaps in the pathophysiology of NP exist because of the effect of sex and the different models used to study the condition, pharmacology-based comparative studies using microglia-specific tools are warranted to uncover the comprehensive mechanisms of NP.

## Data Availability Statement

The raw data supporting the conclusions of this article will be made available by the authors, without undue reservation, to any qualified researcher.

## Ethics Statement

The animal study was reviewed and approved by Animal Research Committee of Wakayama Medical University.

## Author Contributions

Participated in research design: FS and NK. Conducted experiments: FS, DK, YI, and NK. Performed data analysis: FS, SM, TN, SK, and NK. Wrote or contributed to writing the manuscript: FS, TN, SK, and NK.

## Funding

This study was supported by JSPS KAKENHI Grant Numbers 20K09227, 19K09333, 17K15758, 16K08994, and Smoking Research Foundation.

## Conflict of Interest

The authors declare that the research was conducted in the absence of any commercial or financial relationships that could be construed as a potential conflict of interest.

## References

[B1] Alessandri-Haber N. Dina O. A. Chen X. Levine J. D. (2009). TRPC1 and TRPC6 channels cooperate with TRPV4 to mediate mechanical hyperalgesia and nociceptor sensitization. J. Neurosci. 29 (19), 6217–6228. 10.1523/JNEUROSCI.0893-09.2009 19439599 PMC2726836

[B2] Armbruster B. N. Li X. Pausch M. H. Herlitze S. Roth B. L. (2007). Evolving the lock to fit the key to create a family of G protein-coupled receptors potently activated by an inert ligand. Proc. Natl. Acad. Sci. U. S. A 104 (12), 5163–5168. 10.1073/pnas.0700293104 17360345 PMC1829280

[B3] Boccella S. Guida F. De Logu F. De Gregorio D. Mazzitelli M. Belardo C. (2019). Ketones and pain: unexplored role of hydroxyl carboxylic acid receptor type 2 in the pathophysiology of neuropathic pain. FASEB J. 33 (1), 1062–1073. 10.1096/fj.201801033R 30085883

[B4] Burgos E. Gomez-Nicola D. Pascual D. Martin M. I. Nieto-Sampedro M. Goicoechea C. (2012). Cannabinoid agonist WIN 55,212-2 prevents the development of paclitaxel-induced peripheral neuropathy in rats. Possible involvement of spinal glial cells. Eur. J. Pharmacol. 682 (1-3), 62–72. 10.1016/j.ejphar.2012.02.008 22374260

[B5] Chaplan S. R. Bach F. W. Pogrel J. W. Chung J. M. Yaksh T. L. (1994). Quantitative assessment of tactile allodynia in the rat paw. J. Neurosci. Methods 53 (1), 55–63. 10.1016/0165-0270(94)90144-9 7990513

[B6] Chen G. Zhang Y. Q. Qadri Y. J. Serhan C. N. Ji R. R. (2018). Microglia in Pain: Detrimental and Protective Roles in Pathogenesis and Resolution of Pain. Neuron 100 (6), 1292–1311. 10.1016/j.neuron.2018.11.009 30571942 PMC6312407

[B7] Coleman L. G. Jr. Zou J. Crews F. T. (2020). Microglial depletion and repopulation in brain slice culture normalizes sensitized proinflammatory signaling. J. Neuroinflamm. 17 (1), 27. 10.1186/s12974-019-1678-y PMC6969463 31954398

[B8] Colvin L. A. (2019). Chemotherapy-induced peripheral neuropathy: where are we now? Pain 160 (Suppl 1), S1–S10. 10.1097/j.pain.0000000000001540 31008843 PMC6499732

[B9] Del Rivero T. Fischer R. Yang F. Swanson K. A. Bethea J. R. (2019). Tumor necrosis factor receptor 1 inhibition is therapeutic for neuropathic pain in males but not in females. Pain 160 (4), 922–931. 10.1097/j.pain.0000000000001470 30586024

[B10] Dell’Anno M. T. Caiazzo M. Leo D. Dvoretskova E. Medrihan L. Colasante G. (2014). Remote control of induced dopaminergic neurons in parkinsonian rats. J. Clin. Invest. 124 (7), 3215–3229. 10.1172/JCI74664 24937431 PMC4071410

[B11] Di Cesare Mannelli L. Pacini A. Bonaccini L. Zanardelli M. Mello T. Ghelardini C. (2013). Morphologic features and glial activation in rat oxaliplatin-dependent neuropathic pain. J. Pain 14 (12), 1585–1600. 10.1016/j.jpain.2013.08.002 24135431

[B12] Dixon W. J. (1980). Efficient analysis of experimental observations. Annu. Rev. Pharmacol. Toxicol. 20, 441–462. 10.1146/annurev.pa.20.040180.002301 7387124

[B13] Gomez J. L. Bonaventura J. Lesniak W. Mathews W. B. Sysa-Shah P. Rodriguez L. A. (2017). Chemogenetics revealed: DREADD occupancy and activation via converted clozapine. Science 357 (6350), 503–507. 10.1126/science.aan2475 28774929 PMC7309169

[B14] Grace P. M. Wang X. Strand K. A. Baratta M. V. Zhang Y. Galer E. L. (2018). DREADDed microglia in pain: Implications for spinal inflammatory signaling in male rats. Exp. Neurol. 304, 125–131. 10.1016/j.expneurol.2018.03.005 29530713 PMC5916033

[B15] Greter M. Lelios I. Croxford A. L. (2015). Microglia Versus Myeloid Cell Nomenclature during Brain Inflammation. Front. Immunol. 6, 249. 10.3389/fimmu.2015.00249 26074918 PMC4443742

[B16] Hershman D. L. Lacchetti C. Dworkin R. H. Lavoie Smith E. M. Bleeker J. Cavaletti G. (2014). Prevention and management of chemotherapy-induced peripheral neuropathy in survivors of adult cancers: American Society of Clinical Oncology clinical practice guideline. J. Clin. Oncol. 32 (18), 1941–1967. 10.1200/JCO.2013.54.0914 24733808

[B17] Hwang H. W. Saito Y. Park C. Y. Blachere N. E. Tajima Y. Fak J. J. (2017). cTag-PAPERCLIP Reveals Alternative Polyadenylation Promotes Cell-Type Specific Protein Diversity and Shifts Araf Isoforms with Microglia Activation. Neuron 95 (6), 1334–1349 e1335. 10.1016/j.neuron.2017.08.024 28910620 PMC5637551

[B18] Inoue K. Tsuda M. (2018). Microglia in neuropathic pain: cellular and molecular mechanisms and therapeutic potential. Nat. Rev. Neurosci. 19 (3), 138–152. 10.1038/nrn.2018.2 29416128

[B19] Inyang K. E. Szabo-Pardi T. Wentworth E. McDougal T. A. Dussor G. Burton M. D. (2019). The antidiabetic drug metformin prevents and reverses neuropathic pain and spinal cord microglial activation in male but not female mice. Pharmacol. Res. 139, 1–16. 10.1016/j.phrs.2018.10.027 30391353 PMC6447087

[B20] Jensen T. S. Finnerup N. B. (2014). Allodynia and hyperalgesia in neuropathic pain: clinical manifestations and mechanisms. Lancet Neurol. 13 (9), 924–935. 10.1016/S1474-4422(14)70102-4 25142459

[B21] Ji R. R. Chamessian A. Zhang Y. Q. (2016). Pain regulation by non-neuronal cells and inflammation. Science 354 (6312), 572–577. 10.1126/science.aaf8924 27811267 PMC5488328

[B22] Kawasaki Y. Xu Z. Z. Wang X. Park J. Y. Zhuang Z. Y. Tan P. H. (2008). Distinct roles of matrix metalloproteases in the early- and late-phase development of neuropathic pain. Nat. Med. 14 (3), 331–336. 10.1038/nm1723 18264108 PMC2279180

[B23] Kerckhove N. Collin A. Conde S. Chaleteix C. Pezet D. Balayssac D. (2017). Long-Term Effects, Pathophysiological Mechanisms, and Risk Factors of Chemotherapy-Induced Peripheral Neuropathies: A Comprehensive Literature Review. Front. Pharmacol. 8, 86. 10.3389/fphar.2017.00086 28286483 PMC5323411

[B24] Kiguchi N. Kobayashi D. Saika F. Matsuzaki S. Kishioka S. (2017). Pharmacological Regulation of Neuropathic Pain Driven by Inflammatory Macrophages. Int. J. Mol. Sci. 18 (11). 10.3390/ijms18112296 PMC5713266 29104252

[B25] Kiguchi N. Kobayashi D. Saika F. Matsuzaki S. Kishioka S. (2018). Inhibition of peripheral macrophages by nicotinic acetylcholine receptor agonists suppresses spinal microglial activation and neuropathic pain in mice with peripheral nerve injury. J. Neuroinflamm. 15 (1), 96. 10.1186/s12974-018-1133-5 PMC5872578 29587798

[B26] Kiguchi N. Uta D. Ding H. Uchida H. Saika F. Matsuzaki S. (2020). GRP receptor and AMPA receptor cooperatively regulate itch-responsive neurons in the spinal dorsal horn. Neuropharmacology 170, 108025. 10.1016/j.neuropharm.2020.108025 32142790 PMC7754563

[B27] Koga K. Kanehisa K. Kohro Y. Shiratori-Hayashi M. Tozaki-Saitoh H. Inoue K. (2017). Chemogenetic silencing of GABAergic dorsal horn interneurons induces morphine-resistant spontaneous nocifensive behaviours. Sci. Rep. 7 (1), 4739. 10.1038/s41598-017-04972-3 28680103 PMC5498492

[B28] Ledeboer A. Sloane E. M. Milligan E. D. Frank M. G. Mahony J. H. Maier S. F. (2005). Minocycline attenuates mechanical allodynia and proinflammatory cytokine expression in rat models of pain facilitation. Pain 115 (1-2), 71–83. 10.1016/j.pain.2005.02.009 15836971

[B29] Luo X. Huh Y. Bang S. He Q. Zhang L. Matsuda M. (2019). Macrophage Toll-like Receptor 9 Contributes to Chemotherapy-Induced Neuropathic Pain in Male Mice. J. Neurosci. 39 (35), 6848–6864. 10.1523/JNEUROSCI.3257-18.2019 31270160 PMC6733562

[B30] Mapplebeck J. C. Beggs S. Salter M. W. (2016). Sex differences in pain: a tale of two immune cells. Pain 157 (Suppl 1), S2–S6. 10.1097/j.pain.0000000000000389 26785152

[B31] Mei X. P. Xu H. Xie C. Ren J. Zhou Y. Zhang H. (2011). Post-injury administration of minocycline: an effective treatment for nerve-injury induced neuropathic pain. Neurosci. Res. 70 (3), 305–312. 10.1016/j.neures.2011.03.012 21515316

[B32] Milligan E. D. Watkins L. R. (2009). Pathological and protective roles of glia in chronic pain. Nat. Rev. Neurosci. 10 (1), 23–36. 10.1038/nrn2533 19096368 PMC2752436

[B33] Mogil J. S. (2012). Sex differences in pain and pain inhibition: multiple explanations of a controversial phenomenon. Nat. Rev. Neurosci. 13 (12), 859–866. 10.1038/nrn3360 23165262

[B34] Parkhurst C. N. Yang G. Ninan I. Savas J. N. Yates J. R. 3rd Lafaille J. J. (2013). Microglia promote learning-dependent synapse formation through brain-derived neurotrophic factor. Cell 155 (7), 1596–1609. 10.1016/j.cell.2013.11.030 24360280 PMC4033691

[B35] Peng J. Gu N. Zhou L. Eyo U. B. Murugan M. Gan W. B. (2016). Microglia and monocytes synergistically promote the transition from acute to chronic pain after nerve injury. Nat. Commun. 7, 12029. 10.1038/ncomms12029 27349690 PMC4931235

[B36] Pevida M. Lastra A. Hidalgo A. Baamonde A. Menendez L. (2013). Spinal CCL2 and microglial activation are involved in paclitaxel-evoked cold hyperalgesia. Brain Res. Bull. 95, 21–27. 10.1016/j.brainresbull.2013.03.005 23562605

[B37] Raghavendra V. Tanga F. DeLeo J. A. (2003). Inhibition of microglial activation attenuates the development but not existing hypersensitivity in a rat model of neuropathy. J. Pharmacol. Exp. Ther. 306 (2), 624–630. 10.1124/jpet.103.052407 12734393

[B38] Robinson C. R. Zhang H. Dougherty P. M. (2014). Astrocytes, but not microglia, are activated in oxaliplatin and bortezomib-induced peripheral neuropathy in the rat. Neuroscience 274, 308–317. 10.1016/j.neuroscience.2014.05.051 24905437 PMC4099296

[B39] Rosen S. Ham B. Mogil J. S. (2017). Sex differences in neuroimmunity and pain. J. Neurosci. Res. 95 (1-2), 500–508. 10.1002/jnr.23831 27870397

[B40] Saika F. Kiguchi N. Matsuzaki S. Kobayashi D. Kishioka S. (2019). Inflammatory Macrophages in the Sciatic Nerves Facilitate Neuropathic Pain Associated with Type 2 Diabetes Mellitus. J. Pharmacol. Exp. Ther. 368 (3), 535–544. 10.1124/jpet.118.252668 30602591

[B41] Scholz J. Woolf C. J. (2007). The neuropathic pain triad: neurons, immune cells and glia. Nat. Neurosci. 10 (11), 1361–1368. 10.1038/nn1992 17965656

[B42] Seltzer Z. Dubner R. Shir Y. (1990). A novel behavioral model of neuropathic pain disorders produced in rats by partial sciatic nerve injury. Pain 43 (2), 205–218. 10.1016/0304-3959(90)91074-s 1982347

[B43] Sisignano M. Baron R. Scholich K. Geisslinger G. (2014). Mechanism-based treatment for chemotherapy-induced peripheral neuropathic pain. Nat. Rev. Neurol. 10 (12), 694–707. 10.1038/nrneurol.2014.211 25366108

[B44] Smith B. H. Torrance N. (2012). Epidemiology of neuropathic pain and its impact on quality of life. Curr. Pain Headache Rep. 16 (3), 191–198. 10.1007/s11916-012-0256-0 22395856

[B45] Sorge R. E. Mapplebeck J. C. Rosen S. Beggs S. Taves S. Alexander J. K. (2015). Different immune cells mediate mechanical pain hypersensitivity in male and female mice. Nat. Neurosci. 18 (8), 1081–1083. 10.1038/nn.4053 26120961 PMC4772157

[B46] Taves S. Berta T. Liu D. L. Gan S. Chen G. Kim Y. H. (2016). Spinal inhibition of p38 MAP kinase reduces inflammatory and neuropathic pain in male but not female mice: Sex-dependent microglial signaling in the spinal cord. Brain Behav. Immun. 55, 70–81. 10.1016/j.bbi.2015.10.006 26472019 PMC5502100

[B47] Tawfik V. L. Nutile-McMenemy N. Lacroix-Fralish M. L. Deleo J. A. (2007). Efficacy of propentofylline, a glial modulating agent, on existing mechanical allodynia following peripheral nerve injury. Brain Behav. Immun. 21 (2), 238–246. 10.1016/j.bbi.2006.07.001 16949251

[B48] Thacker M. A. Clark A. K. Marchand F. McMahon S. B. (2007). Pathophysiology of peripheral neuropathic pain: immune cells and molecules. Anesth. Analg. 105 (3), 838–847. 10.1213/01.ane.0000275190.42912.37 17717248

[B49] Trang T. Beggs S. Wan X. Salter M. W. (2009). P2X4-receptor-mediated synthesis and release of brain-derived neurotrophic factor in microglia is dependent on calcium and p38-mitogen-activated protein kinase activation. J. Neurosci. 29 (11), 3518–3528. 10.1523/JNEUROSCI.5714-08.2009 19295157 PMC3589565

[B50] Urban D. J. Roth B. L. (2015). DREADDs (designer receptors exclusively activated by designer drugs): chemogenetic tools with therapeutic utility. Annu. Rev. Pharmacol. Toxicol. 55, 399–417. 10.1146/annurev-pharmtox-010814-124803 25292433

[B51] van Hecke O. Austin S. K. Khan R. A. Smith B. H. Torrance N. (2014). Neuropathic pain in the general population: a systematic review of epidemiological studies. Pain 155 (4), 654–662. 10.1016/j.pain.2013.11.013 24291734

[B52] Yu X. Liu H. Hamel K. A. Morvan M. G. Yu S. Leff J. (2020). Dorsal root ganglion macrophages contribute to both the initiation and persistence of neuropathic pain. Nat. Commun. 11 (1), 264. 10.1038/s41467-019-13839-2 31937758 PMC6959328

[B53] Zhang B. Zou J. Han L. Beeler B. Friedman J. L. Griffin E. (2018). The specificity and role of microglia in epileptogenesis in mouse models of tuberous sclerosis complex. Epilepsia 59 (9), 1796–1806. 10.1111/epi.14526 30079598 PMC6121723

[B54] Zhang T. Yanagida J. Kamii H. Wada S. Domoto M. Sasase H. (2020). Glutamatergic neurons in the medial prefrontal cortex mediate the formation and retrieval of cocaine-associated memories in mice. Addict. Biol. 25 (1), e12723. 10.1111/adb.12723 30734456

[B55] Zhao H. Alam A. Chen Q. Eusman M. A. Pal A. Eguchi S. (2017). The role of microglia in the pathobiology of neuropathic pain development: what do we know? Br. J. Anaesth. 118 (4), 504–516. 10.1093/bja/aex006 28403399

[B56] Zhao X. F. Alam M. M. Liao Y. Huang T. Mathur R. Zhu X. (2019). Targeting Microglia Using Cx3cr1-Cre Lines: Revisiting the Specificity. eNeuro 6 (4). 10.1523/ENEURO.0114-19.2019 PMC6620394 31201215

[B57] Zhu H. Aryal D. K. Olsen R. H. Urban D. J. Swearingen A. Forbes S. (2016). Cre-dependent DREADD (Designer Receptors Exclusively Activated by Designer Drugs) mice. Genesis 54 (8), 439–446. 10.1002/dvg.22949 27194399 PMC4990490

