# Supplementary material for: Chemogenetic Regulation of CX3CR1-Expressing Microglia Using Gi-DREADD Exerts Sex-Dependent Anti-Allodynic Effects in Mouse Models of Neuropathic Pain
Source: Front Pharmacol. 2020 Jun 19;11:925. doi: 10.3389/fphar.2020.00925 (PMC7318895; doi:10.3389/fphar.2020.00925)
Supplement: Supplementary file 1 [file DataSheet_1.pdf]

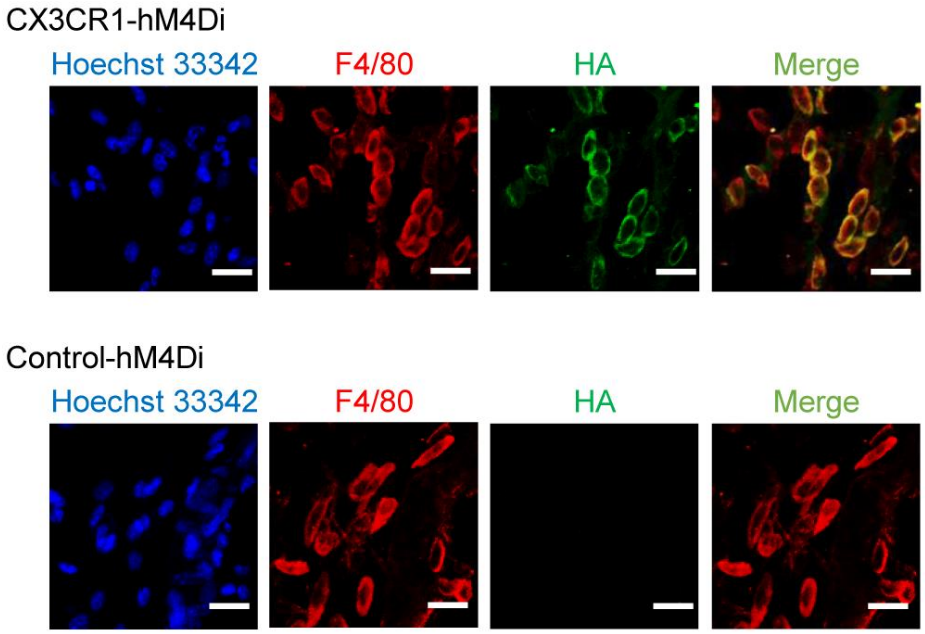

Supplementary Figure 1. hM4Di expression in peripheral macrophages of CX3CR1-hM4Di mice. Male CX3CR1-hM4Di and Control-hM4Di mice were subjected to PSL, and the injured sciatic nerve (SCN) was dissected on day 7 after PSL. Expression of HA-tagged hM4Di in F4/80$^+$ macrophages of CX3CR1-hM4Di, but not Control-hM4Di mice, was visualized by immunohistochemistry. Scale bars = 10 μm.

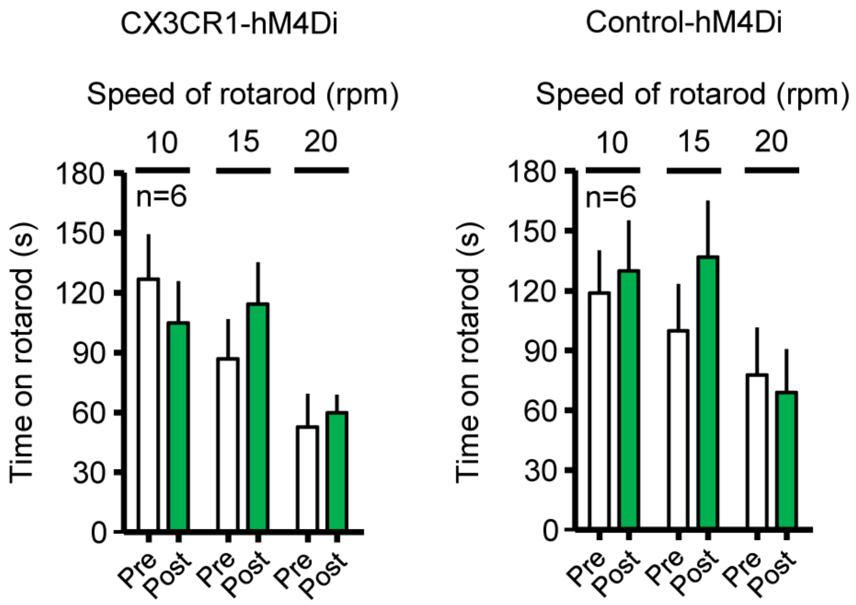

Supplementary Figure 2. CNO effect on motor function in CX3CR1-hM4Di and Control-hM4Di mice. Male CX3CR1-hM4Di and Control-hM4Di mice were placed on the Rotarod before (Pre) and 24 h after administration (Post) of CNO (10 mg/kg, i.p.), at different speeds (10, 15 and 20 rpm). The graphs present the time that the mice spent on the Rotarod, on each observation, before falling. Data are presented as mean ± S.E.M. n = 6.
